# Characterization of the integrated gut microbiota and metabolite profiles in osteoporosis patients with different traditional Chinese medicine syndromes

**DOI:** 10.3389/fmicb.2025.1663716

**Published:** 2025-10-22

**Authors:** Zixiang Geng, Long Yuan, Dihang See, Yongfang Zhao

**Affiliations:** ^1^Shi's Center of Orthopedics and Traumatology, Shuguang Hospital Affiliated to Shanghai University of Traditional Chinese Medicine, Shanghai, China; ^2^Institute of Traumatology and Orthopedics, Shanghai Academy of Traditional Chinese Medicine, Shanghai, China

**Keywords:** gut microbiota, osteoporosis, traditional Chinese medicine, kidney Yang deficiency syndrome, spleen-kidney Yang deficiency

## Abstract

**Introduction:**

This study aims to investigate whether the Traditional Chinese Medicine (TCM) classification of osteoporosis corresponds to specific gut microbial and metabolic profiles, thereby providing a microbiological basis for TCM syndrome differentiation.

**Methods:**

Body composition was assessed using dual-energy X-ray absorptiometry in healthy elderly controls and osteoporosis patients categorized by TCM subtype. Gut microbiota composition and metabolite profiles were analyzed via 16S rRNA gene sequencing and liquid chromatography-tandem mass spectrometry (LC-MS/MS), respectively.

**Results:**

The gut microbiota dysbiosis index was significantly elevated in osteoporosis patients compared to healthy controls, with the highest levels observed in the spleen-kidney Yang deficiency subtype. Distinct microbial signatures were identified: *Intestinibacter* and *Phascolarctobacterium* were significantly enriched in kidney Yang deficiency osteoporosis, while *Olsenella* was markedly increased in spleen-kidney Yang deficiency osteoporosis. Correlation analyses revealed significant associations between these microbial markers and clinical parameters: *Intestinibacter* and *Phascolarctobacterium* abundances negatively correlated with bone mineral density at multiple skeletal sites, whereas *Olsenella* levels were negatively associated with appendicular skeletal muscle index. Importantly, microbial metabolic pathways differed between TCM subtypes, with kidney Yang deficiency associated with vitamin D metabolism and spleen-kidney Yang deficiency linked to lipid metabolism.

**Conclusion:**

TCM classification captures meaningful biological heterogeneity in osteoporosis, reflected in distinct microbiome and metabolic signatures. These findings provide a microbiological basis for TCM syndrome differentiation and may inform personalized approaches to osteoporosis diagnosis and treatment.

## 1 Introduction

Osteoporosis (OP) is the most common metabolic bone disease, characterized by the destruction of bone microstructure, reduction of bone biomechanical properties, and increased brittleness, leading to recurrent fractures (Kubi et al., 2024; Ensrud and Crandall, 2017). OP frequently occurs in postmenopausal women, with some individuals also experiencing sarcopenia or high body fat, resulting in a significantly higher risk of falls and fractures compared to individuals with OP without accompanying symptoms. We refer to this condition as dysmobility syndrome based on OP (ODS; Geng et al., 2024). ODS refers to a cluster of symptoms that frequently accompany osteoporosis, including reduced muscle strength, decreased muscle mass, elevated body fat, and a history of falls. This concept draws a parallel to the notion of metabolic syndrome. The introduction of ODS emphasizes the importance of assessing the comprehensive mobility and functional capacity of osteoporosis patients, since the risk of falls and fractures is influenced not only by low bone mineral density but also by impaired muscle function, altered body composition, and prior fall experiences. Therefore, the treatment of OP increasingly emphasizes comprehensive management of muscle-bone-fat, making further classification of OP beneficial for personalized therapy. In Traditional Chinese Medicine (TCM), kidney Yang deficiency and spleen-kidney Yang deficiency are common syndromes associated with OP. The term “spleen-kidney” does not refer to traditional organs in the classical sense but rather to certain systems that perform specific functions; for instance, “spleen” encompasses the digestive system, including the stomach, intestines, and spleen, with spleen deficiency often indicating digestive disorders. Previous studies have indicated that spleen-kidney deficiency is a key etiological factor in osteoporosis (An et al., 2025). Patients with this deficiency pattern typically present with symptoms such as digestive disorders, sallow complexion, muscle wasting, and general debility, which resemble the manifestations of ODS. However, the scientific connotations of these syndromes remain unclear, and their intrinsic relationship with POP warrants further exploration.

According to TCM theory, the spleen governs the transportation and transformation of nutrients, making it closely involved in the absorption and metabolism of essential substances. This concept shows a remarkable convergence with the role of the gut microbiota in modern medicine, which is similarly essential for nutrient processing, metabolic regulation, and maintaining systemic homeostasis. Numerous studies indicate that the gut microbiota serves as the material basis for the spleen, and the spleen's transformative functions are closely related to the diversity and functionality of the gut microbiota (Li et al., 2025; Wang et al., 2023; Ma et al., 2021). Emerging evidence suggests that gut microbiota dysbiosis mediates, at least in part, the pathogenesis of osteoporosis. In POP, the gut microbiota can modulate bone metabolism through various mechanisms via the gut-bone axis (Zhang et al., 2024b). For instance, Lactobacillus and Bifidobacterium can enhance the absorption of minerals such as calcium and phosphorus, thereby increasing bone density, while short-chain fatty acid-producing bacteria can inhibit osteoclast-mediated bone resorption through the production of butyrate (Lucas et al., 2018). Furthermore, the association between gut microbiota and muscle is even more pronounced. The gut microbiota can influence the balance of muscle protein synthesis and degradation by modulating inflammatory responses, insulin sensitivity, and energy metabolism, thereby playing a role in the development and progression of sarcopenia (Giron et al., 2022). From the perspective of TCM, this phenomenon resonates with the theory that “the spleen governs the muscles.” Spleen deficiency may lead to malnourishment of the muscles, a concept that aligns closely with muscle metabolic abnormalities induced by gut microbiota dysbiosis. Therefore, investigating the gut microbiota and its metabolites not only helps to elucidate the modern biological basis of osteoporosis with spleen-kidney Yang deficiency but also holds promise for identifying specific microbial biomarkers and their functional pathways related to this TCM syndrome. This approach provides a novel perspective for understanding its biological underpinnings.

Therefore, in this study, we elucidated the microbial and metabolic characteristics associated with different types of OP through gut microbiota diversity and metabolomics, and we initially explored the intrinsic relationship within the gut microbiota-metabolite-muscle-bone-fat metabolic axis.

## 2 Materials and methods

### 2.1 Study subjects

This study is an exploratory research with a small sample size. Considering the prevalence of OP in women and to avoid heterogeneity caused by gender differences, we included only female participants in this study. A total of 90 female subjects were enrolled, comprising 30 elderly participants without OP, 30 women with kidney Yang deficiency type OP, and 30 women with spleen-kidney Yang deficiency type OP. This study was approved by the Ethics Committee of Shanghai University of Traditional Chinese Medicine (2023-1-5-03).

### 2.2 Diagnostic criteria

The diagnosis of OP can be made if any one of the following three criteria is met: (1) Fragility fracture of the hip or vertebral body; (2) Fragility fracture of the proximal humerus, pelvis, or distal forearm, with bone density measurements showing −2.5 < *T*-score < −1.0; (3) Bone density measurements showing *T*-score ≤ −2.5.

### 2.3 Traditional Chinese medicine diagnostic criteria

Low back pain is a mandatory symptom, and the diagnosis can be established if it is accompanied by any two other symptoms and supported by tongue and pulse examination. The specific diagnostic details are as follows: Kidney Yang Deficiency: Symptoms include cold pain in the lower back, soreness, weakness, significant hunching or bending, limited mobility, aversion to cold and preference for warmth, worsening pain in cold conditions (especially in the lower limbs), frequent urination, chronic diarrhea, or edema (with swelling more pronounced below the waist), tender and pale tongue, white coating, and deep, thin, or deep, wiry pulse. Spleen-Kidney Yang Deficiency: Symptoms include cold pain in the lower back and hips, soreness and weakness in the lower back and knees, significant hunching, aversion to cold and preference for warmth, pallor, early morning diarrhea, clear watery stool with bowel movements, difficulty in urination, facial swelling and limb edema, or abdominal distension, with a pale, swollen tongue, white, slippery coating, and weak or slow pulse.

### 2.4 Bone mineral density and body composition analysis

Bone mineral density (BMD) and body composition were assessed using DXA (Dual-Energy X-ray Absorptiometry). For body composition metrics, appendicular skeletal muscle index (ASMI) of less than 5.4 in women is diagnosed as low muscle mass, while a body fat percentage (fat%) greater than 40% is diagnosed as high body fat.

### 2.5 Muscle strength assessment

Handgrip strength (HGS) is a key indicator for evaluating muscle strength and is considered a critical parameter for sarcopenia diagnosis by the European Working Group on Sarcopenia in Older People (EWGSOP) and the Asian Working Group for Sarcopenia (AWGS; Chen et al., 2020; Cruz-Jentoft et al., 2019). To assess muscle strength, HGS is measured using an electronic hand dynamometer, testing the dominant hand. The strength is measured three times, with a 60 s interval between each measurement, and the maximum value is recorded. A maximum HGS of less than 18 kg is diagnosed as low muscle strength.

### 2.6 Six-meter walk test

Walking speed is an important indicator for assessing an individual's ability to perform daily activities and lower limb muscle function, and it aids in the diagnosis of sarcopenia. A decrease in walking speed (< 1.0 m/s) is a significant marker of diminished muscle strength and function.

### 2.7 Five times sit-to-stand test

A sit-to-stand test time of greater than 12 s for five repetitions is diagnosed as a decline in physical function.

### 2.8 Calf circumference measurement

Calf circumference is a simple proxy indicator for muscle mass and is highly correlated with the amount of appendicular skeletal muscle measured by DXA. Calf circumference is measured with a non-elastic fiber measuring tape (with a precision of 1 mm). A measurement of less than 34 cm for men and less than 33 cm for women is considered indicative of a decline in muscle mass.

### 2.9 Fracture risk assessment

Fracture risk is assessed using the FRAX tool recommended by the World Health Organization (https://www.sheffield.ac.uk/FRAX/), which outputs a 10-year fracture probability, including the probability of major osteoporotic (MO) and hip fracture (HF).

### 2.10 Fall risk assessment

The fall risk of participants is evaluated using the community fall risk assessment tool (FROP-Com) recommended by Australia. The total score is 60 points; the higher the score, the greater the risk. A FROP-Com score of ≥12 points is defined as high fall risk.

### 2.11 Mini nutritional assessment

The Mini Nutritional Assessment (MNA) is an internationally recognized tool for screening the nutritional status of the elderly, designed to quickly identify malnutrition and risk. This study utilized the short form of the assessment (MNA-SF), which comprises six items: weight loss (in the past 3 months), BMI, disease or acute stress status, functional ability, neuropsychological problems (dementia, depression), and changes in dietary intake (in the past 3 months). The total score of the scale is 14 points; a higher score indicates better nutritional status. A score of ≤ 7 points indicates malnutrition, a score of 8–11 points indicates a risk of malnutrition, and a score of ≥12 points indicates normal nutrition.

### 2.12 Gut microbiota diversity and metabolomics

Fecal samples were stored at −80 °C and sent to Shanghai Meiji Biomedical Technology Co., Ltd. for analysis. Detailed information can be found in the Supplementary methods and materials.

### 2.13 Statistical analysis

Statistical analyses were performed using GraphPad Prism 7. Continuous data were described using means ± standard deviations, while categorical data were described using frequencies (percentages). For intergroup comparisons, data that followed a normal distribution were analyzed using *t*-tests or one-way ANOVA, while data that did not meet the normal distribution were analyzed using the Wilcoxon rank-sum test. The chi-square test was used for comparison of proportions. A *P*-value of < 0.05 was considered statistically significant.

## 3 Results

### 3.1 Baseline data

We categorized OP into two types: kidney Yang deficiency type (OP-K) and spleen-kidney Yang deficiency type (OP-S), with average ages of 69.87 and 69.73 years, respectively. Compared to elderly women without OP, women with kidney Yang deficiency type OP and those with spleen-kidney Yang deficiency type OP exhibited significantly lower height, weight, and BMI, with the spleen-kidney Yang deficiency type showing even lower values (*P* < 0.05). In terms of muscle mass and functional assessment, women with OP demonstrated significantly reduced maximum grip strength, prolonged time to complete five sit-to-stand transitions, and slower 6-meter walking speed compared to elderly women without OP. Furthermore, women with spleen-kidney Yang deficiency type OP had lower maximum grip strength, longer sit-to-stand times, and slower walking speeds compared to those with kidney Yang deficiency type OP. Among women with kidney Yang deficiency type OP, 16.67% had low grip strength, 23.33% had poor physical function, and 46.67% had reduced walking speed, whereas these rates were significantly higher at 33.33%, 33.33%, and 80%, respectively, in women with spleen-kidney Yang deficiency type OP. Regarding BMD, women with spleen-kidney Yang deficiency type OP exhibited lower densities in L1–L4, femoral neck, and total hip compared to those with kidney Yang deficiency type OP, along with higher clinical symptom scores. In terms of fat and muscle mass, both elderly women without OP and those with OP had elevated body fat levels at 39% and 38%, respectively, and there were no significant changes in the ratio of abdominal to gluteal fat or visceral fat mass. Compared to elderly women without OP, both groups of women with OP had reduced lean body mass, ASMI, and calf circumference, with women in the spleen-kidney Yang deficiency type having lower values than those in the kidney Yang deficiency type. In terms of the risk of falls and fractures, both groups of women with OP showed increased risks of falls, major fractures, and hip fractures compared to elderly women without OP, with the spleen-kidney Yang deficiency type showing a greater increase. The rate of previous fractures among both groups of women with OP reached 60%, compared to 20% in elderly women without OP. Regarding body composition, both groups of women with OP exhibited significant reductions in total body fat, muscle, and bone mineral content, with the spleen-kidney Yang deficiency type showing greater decreases. In the evaluation of nutrition and quality of life, women with spleen-kidney Yang deficiency type OP had significantly lower nutrition scores (*P* < 0.01), suggesting the possibility of poor nutritional status. Compared to elderly women without OP, both groups of women with OP showed significantly lower scores in social functioning, and women with spleen-kidney Yang deficiency type OP had significantly lower energy scores compared to those with kidney Yang deficiency type OP (Table 1).

**Table 1:** Baseline data.

**Group/variables**	**Totle (*n* = 90)**	**NP_O (*n* = 30)**	**OP_K (*n* = 30)**	**OP_S (*n* = 30)**	**Statistic**	***P***
Age	69.32 ± 4.74	68.37 ± 4.73	69.87 ± 4.34	69.73 ± 5.13	*F* = 0.92	0.403
Height (cm)	154.84 ± 4.76	156.48 ± 3.95	154.87 ± 3.86	153.17 ± 5.77	*F* = 3.88	0.024
Weight (kg)	55.73 ± 7.69	59.68 ± 7.03	56.15 ± 6.18	51.37 ± 7.60	*F* = 10.78	< 0.001
BMI	23.25 ± 2.76	24.36 ± 2.61	23.41 ± 2.42	22.00 ± 2.80	*F* = 6.17	0.003
HGS (kg)	21.26 ± 3.83	22.72 ± 3.41	21.98 ± 4.26	19.07 ± 2.73	*F* = 9.01	< 0.001
Low grip strength	17 (18.89)	2 (6.67)	5 (16.67)	10 (33.33)	X^2^ = 7.11	0.029
5-STS (s)	10.48 ± 2.84	10.16 ± 3.25	10.23 ± 2.67	11.05 ± 2.54	*F* = 0.91	0.408
Low physical function	25 (27.78)	8 (26.67)	7 (23.33)	10 (33.33)	X^2^ = 0.78	0.679
6 m-speed (m/s)	1.02 ± 0.20	1.06 ± 0.22	1.07 ± 0.20	0.94 ± 0.12	*F* = 4.76	0.011
Low speed	52 (57.78)	14 (46.67)	14 (46.67)	24 (80.00)	X^2^ = 9.11	0.011
L1–L4 BMD (g/cm^3^)	0.93 ± 0.18	1.08 ± 0.15	0.87 ± 0.13	0.82 ± 0.14	*F* = 30.24	< 0.001
Neck BMD (g/cm^3^)	0.73 ± 0.13	0.85 ± 0.11	0.69 ± 0.09	0.65 ± 0.09	*F* = 34.85	< 0.001
Total BMD (g/cm^3^)	0.79 ± 0.14	0.93 ± 0.11	0.73 ± 0.09	0.72 ± 0.09	*F* = 48.35	< 0.001
Fat (%)	0.39 ± 0.05	0.39 ± 0.05	0.38 ± 0.03	0.39 ± 0.06	*F* = 0.11	0.895
High body fat	31 (34.44)	11 (36.67)	8 (26.67)	12 (40.00)	X^2^ = 1.28	0.527
Android (%)	43.71 ± 7.75	43.68 ± 8.05	44.20 ± 4.65	43.26 ± 9.87	*F* = 0.11	0.897
Gynoid (%)	37.41 ± 4.70	37.71 ± 4.01	37.01 ± 3.81	37.52 ± 6.06	*F* = 0.18	0.838
A/G	1.17 ± 0.18	1.16 ± 0.18	1.20 ± 0.16	1.15 ± 0.20	*F* = 0.84	0.435
Visceral fat (g)	900.98 ± 391.58	975.40 ± 420.08	871.03 ± 274.38	856.50 ± 458.39	*F* = 0.82	0.444
Lean body mass (g)	35318.38 ± 3961.54	37946.47 ± 3645.83	35677.87 ± 3086.77	32330.80 ± 2971.79	*F* = 22.69	< 0.001
Calf circumference (cm)	33.65 ± 2.30	34.58 ± 1.79	33.95 ± 2.16	32.42 ± 2.40	*F* = 8.10	< 0.001
ASMI	5.88 ± 0.70	6.23 ± 0.69	6.06 ± 0.58	5.36 ± 0.50	*F* = 17.64	< 0.001
Low muscle mass	23 (25.56)	3 (10.00)	5 (16.67)	15 (50.00)	X^2^ = 14.48	< 0.001
MO	8.61 ± 6.03	5.00 ± 2.55	9.96 ± 4.51	10.87 ± 8.01	*F* = 9.89	< 0.001
HF	3.70 ± 4.79	1.17 ± 0.93	4.36 ± 3.44	5.58 ± 6.85	*F* = 7.83	< 0.001
Fall risk	5.46 ± 3.98	5.30 ± 3.45	5.43 ± 4.27	5.63 ± 4.28	*F* = 0.05	0.949
History of fracture	42 (46.67)	6 (20.00)	18 (60.00)	18 (60.00)	X^2^ = 12.86	0.002
Total body fat (g)	21364.96 ± 4795.98	23073.53 ± 4673.25	21192.13 ± 3632.68	19829.20 ± 5483.08	*F* = 3.67	0.030
Total body muscle (g)	33543.74 ± 3773.52	35931.67 ± 3519.92	33966.13 ± 3000.57	30733.43 ± 2837.90	*F* = 21.06	< 0.001
Total body bone mineral content (g)	1775.01 ± 259.63	2013.30 ± 220.54	1710.37 ± 150.68	1601.37 ± 203.19	*F* = 36.40	< 0.001
MNA	12.98 ± 1.41	13.37 ± 1.07	13.27 ± 1.11	12.30 ± 1.74	*F* = 5.76	0.004

BMI, Body Mass Index; HGS, Handgrip Strength; 5-STS, Five Times Sit-to-Stand Test; BMD, Bone mineral density; A/G, Android/Gynoid; ASMI, Appendicular Skeletal Muscle Index; MO, Major Osteoporotic; HF, Hip Fracture; MNA, Mini Nutritional Assessment.

### 3.2 Analysis of gut microbiota diversity in different types of OP

**Figure 1:**
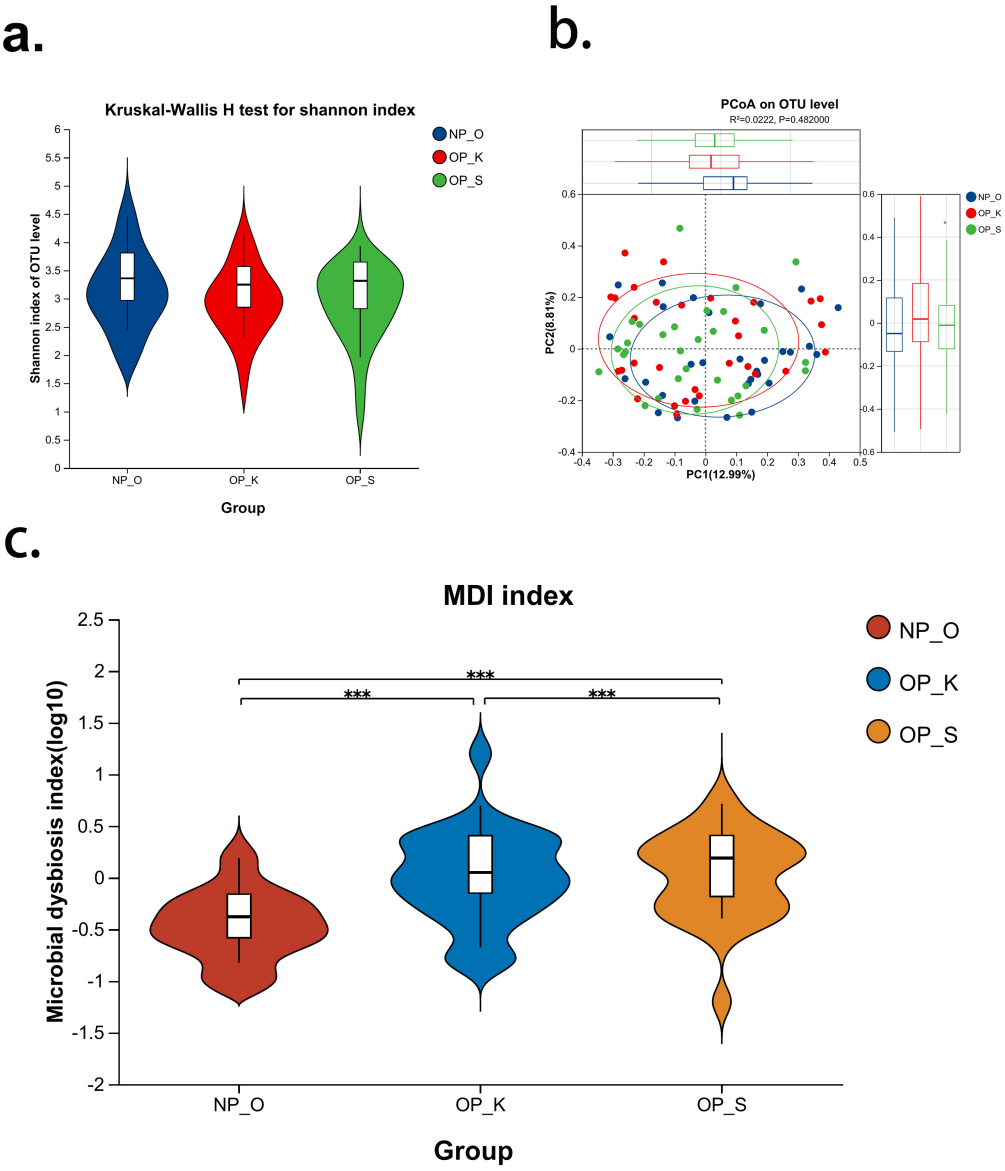
Analysis of gut microbiota diversity in patients with different type of OP. **(a)** Alpha diversity index—Shannon; **(b)** Beta diversity index—PCoA; **(c)** Gut microbiota dysbiosis index—MDI; NP_O, Elderly women without OP; OP_K, Women with kidney Yang deficiency type OP; OP_S, Women with spleen-kidney Yang deficiency type OP; ***P < 0.001.

The results of alpha diversity analysis (Figure 1a) indicate that there is no significant difference in the Shannon index among the three groups, suggesting that the microbial abundance and diversity at the operational taxonomic unit (OTU) level are not significantly different between women with kidney Yang deficiency type OP, spleen-kidney Yang deficiency type OP, and elderly women without OP. Principal coordinates analysis (PCoA; Figure 1b) shows that the microbial compositions of the three groups are similar. The microbial characterization analysis (Figure 1c) reveals that the dysbiosis index in women with spleen-kidney Yang deficiency type OP is significantly higher than that in women with kidney Yang deficiency type and elderly women without OP (*P* < 0.001). These results on microbial diversity suggest that there are no significant differences in the overall composition and diversity of gut microbiota among women with different types of OP and elderly women without OP. However, the dysbiosis index (MDI) is significantly elevated in women with spleen-kidney Yang deficiency type OP, thus indicating the need for further analysis of microbial differences at various taxonomic levels.

### 3.3 Characteristics of gut microbiota at the phylum level in different types of OP

**Figure 2:**
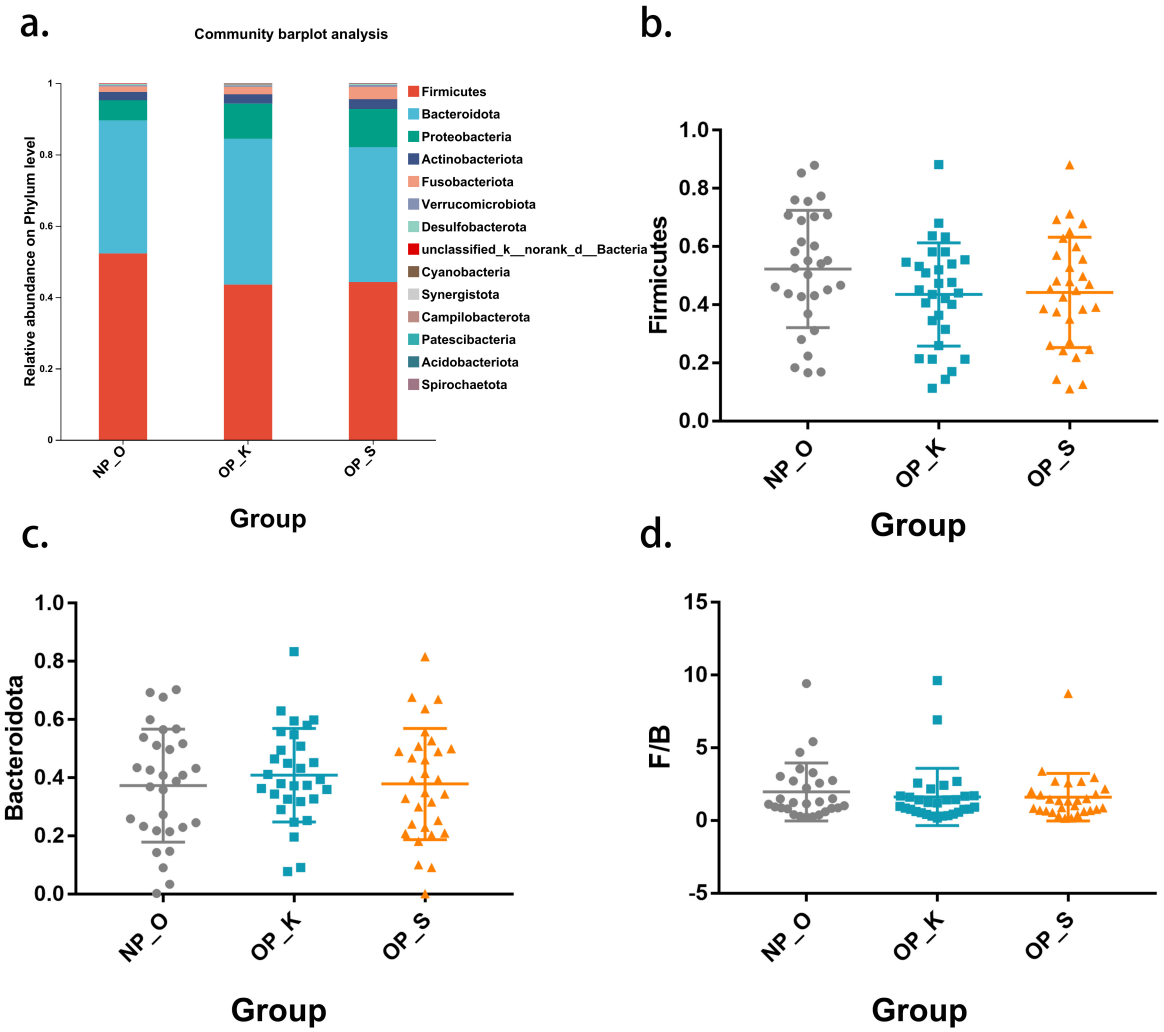
Phylum level microbiota characteristics in different type of OP. **(a)** Composition of the gut microbiota at the phylum level. **(b)** Differential analysis of Firmicutes. **(c)** Differential analysis of Bacteroidota. **(d)** Differential analysis of the F/B ratio.

The microbial composition at the phylum level in the three groups is primarily dominated by Firmicutes and Bacteroidota (Figure 2a). The proportion of Firmicutes in women with OP is lower than that in elderly women without OP (spleen-kidney Yang deficiency type: 44.23%; kidney Yang deficiency type: 43.53%; elderly women without OP: 52.28%). There are no significant differences in the relative abundances of Firmicutes and Bacteroidota among the three groups (Figures 2b, c). The ratio of Firmicutes to Bacteroidota (F/B) is commonly used to characterize dysbiosis and disease states; however, there are no significant differences in F/B among patients with OP (Figure 2d). These results suggest that there are no significant differences in gut microbiota at the phylum level among the three groups, indicating the need for further clarification of microbial characteristics at the genus level.

### 3.4 Characteristics of gut microbiota at the genus level in different types of OP

**Figure 3:**
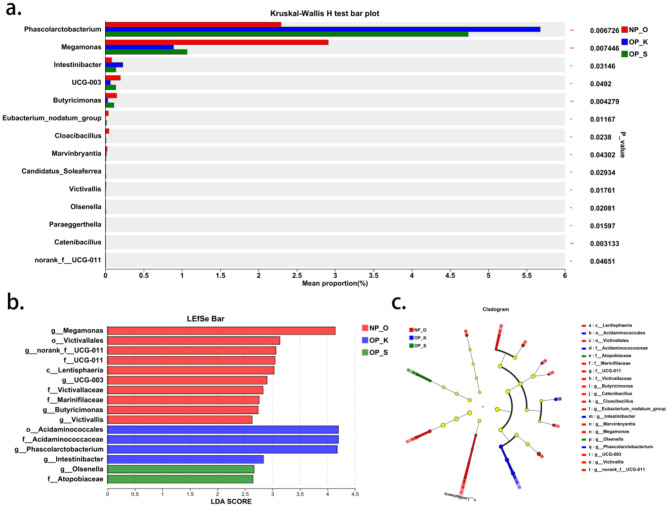
Genus level microbiota characteristics in different types of OP. **(a)** Differential analysis of gut microbiota at the genus level. **(b, c)** Characteristic genera in different types of OP.

At the genus level, there are 14 differential microbial taxa shared among the three groups (Figure 3a). Linear discriminant analysis effect size (Lefse) results indicate that the genera *Intestinibacter* and *Phascolarctobacterium*, along with their respective families *Acidaminococcaceae* and order *Acidaminococcales*, are significantly enriched in women with kidney Yang deficiency type OP. Conversely, the genus *Olsenella* and its associated family *Atopobiaceae* are significantly enriched in women with spleen-kidney Yang deficiency type OP, while *Megamonas*, the genus *Butyricimonas*, and the genus *Victivallis* are significantly enriched in elderly women without OP (Figures 3b, c). These findings suggest that the core characteristic microbial taxa for women with kidney Yang deficiency type OP are *Intestinibacter* and *Phascolarctobacterium*, while the core characteristic microbial taxon for women with spleen-kidney Yang deficiency type OP is *Olsenella*.

### 3.5 Analysis of the correlation between gut microbiota and muscle-bone-fat

**Figure 4:**
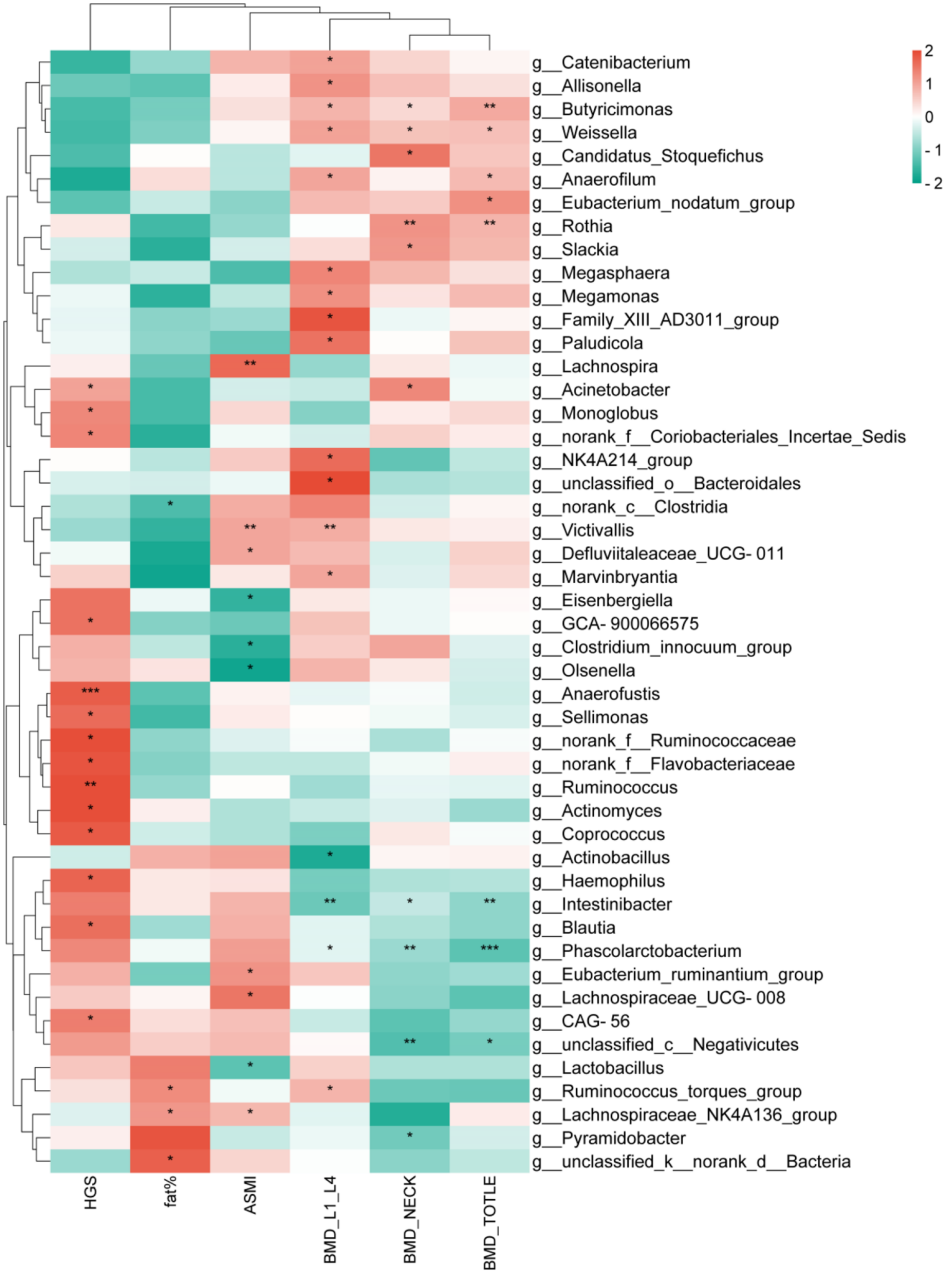
Correlation analysis between gut microbiota and clinical factors. *P < 0.05,**P < 0.01,***P < 0.001.

A correlation analysis was conducted between the top 200 abundant genera and HGS, body fat percentage (fat%), ASMI, BMD, revealing that 48 genera were correlated with the aforementioned clinical factors. In the core characteristic microbiota of women with kidney Yang deficiency type OP, *Intestinibacter* and *Phascolarctobacterium* were negatively correlated with L1–L4, neck, and total BMD. In the core characteristic microbiota of women with spleen-kidney Yang deficiency type OP, *Olsenella* was negatively correlated with ASMI. Given the significantly reduced MNA score observed in patients with spleen-kidney Yang deficiency type OP, we hypothesized that nutritional status might mediate the relationship between gut microbiota and the clinical phenotype. Correlation analysis revealed that the abundance of *Olsenella* was significantly and negatively correlated with MNA scores (Supplementary Figure S1). Furthermore, mediation analysis demonstrated that MNA plays a mediating role in the effect of *Olsenella* on the phenotype of spleen-kidney Yang deficiency type OP (Supplementary Figure S2). Among other differential genera, *Megamonas* was positively correlated with L1–L4 BMD, *Butyricimonas* was positively correlated with L1–L4, neck, and total, and the genus *Victivallis* was positively correlated with L1–L4 BMD and ASMI (Figure 4).

### 3.6 Differential metabolites of gut microbiota in different types of OP

**Figure 5:**
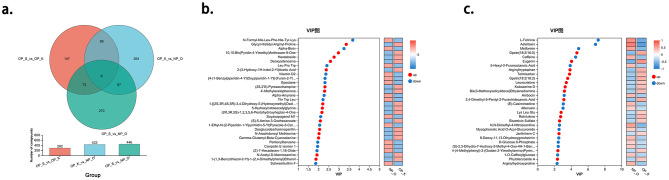
Analysis of Characteristic Metabolic Profiles in Different Types of OP. **(a)** Establishment of characteristic metabolic profiles for different types of OP. **(b)** Characteristic metabolites of kidney Yang deficiency type OP. **(c)** Characteristic metabolites of spleen-kidney Yang deficiency type OP.

Among the metabolites, compared to elderly women without OP, women with kidney Yang deficiency type OP exhibited a total of 433 differential metabolites, including 272 upregulated and 161 downregulated. Likewise, women with spleen-kidney Yang deficiency type OP compared to elderly women without OP had a total of 446 differential metabolites, with 302 upregulated and 144 downregulated. When comparing women with spleen-kidney Yang deficiency type OP to those with kidney Yang deficiency type OP, there were 292 differential metabolites, with 137 upregulated and 155 downregulated. By intersecting the three sets of differential metabolites, we defined the intersection of the OP_S_vs_OP_K differential set and the OP_K_vs_NP_O differential set as the metabolic characteristic set for kidney Yang deficiency type OP. Similarly, the intersection of the OP_S_vs_OP_K differential set and the OP_S_vs_NP_O differential set was defined as the metabolic characteristic set for spleen-kidney Yang deficiency type OP. Among these, six metabolites were found in both characteristic sets: Metformin, Asteltoxin, L-Felinine, Eugenin, Gpetn (18:2/16:0), and Gpetn (18:2/18:2). Compared to the other two groups, Metformin, Asteltoxin, and L-Felinine were higher in the spleen-kidney Yang deficiency type OP group, while Eugenin, Gpetn (18:2/16:0), and Gpetn (18:2/18:2) were lower. Therefore, all six metabolites were included in the differential metabolic characteristic set for spleen-kidney Yang deficiency type OP, rather than the set for kidney Yang deficiency type OP (Figure 5a).

Using OPLS-DA as a supervised model, we performed a validation through 7-fold cross-validation to examine the prediction of different changes in paired samples. The Variable Importance in Projection (VIP) analysis of the first principal component was employed to identify important metabolites that contribute to classification. In the metabolic characteristic set for kidney Yang deficiency type OP, the top 10 metabolites, in order, are N-Formyl-Nle-Leu-Phe-Nle-Tyr-Lys, Glycyl-Histidyl-Arginyl-Proline, Alpha-Bixin, 10,10-Bis(Pyridin-4-Ylmethyl)Anthracen-9-One, Hastatoside, Deoxyadenosine, LeuProTrp, 2-(3-Hydroxy-1H-Indol-2-Yl)Acetic Acid, Vitamin D2, and [4-(1-Benzylpiperidin-4-Yl)Oxypiperidin-1-Yl]-(Furan-2-Yl)Methanone. Among these, N-Formyl-Nle-Leu-Phe-Nle-Tyr-Lys, Alpha-Bixin, LeuProTrp, vitamin D2, and [4-(1-Benzylpiperidin-4-Yl)Oxypiperidin-1-Yl]-(Furan-2-Yl)Methanone are considered downregulated metabolites, while glycyl-histidyl-arginyl-proline, 10,10-Bis(Pyridin-4-Ylmethyl)Anthracen-9-One, Hastatoside, Deoxyadenosine, and 2-(3-Hydroxy-1H-Indol-2-Yl)Acetic Acid are considered upregulated metabolites (Figure 5b).

In the metabolic characteristic set for spleen-kidney Yang deficiency type OP, the top 10 metabolites, in order, are L-Felinine, Asteltoxin, Metformin, Gpetn (18:2/16:0), Caffeine, Eugenin, 5-Hexyl-2-Furanoctanoic Acid, Arginyltryptophan, Telmisartan, and Gpetn (18:2/18:2). Among these, L-Felinine, Asteltoxin, Metformin, Caffeine, and 5-Hexyl-2-Furanoctanoic Acid are considered downregulated metabolites, while Gpetn (18:2/16:0), Eugenin, Arginyltryptophan, Telmisartan, and Gpetn (18:2/18:2) are considered upregulated metabolites (Figure 5c).

### 3.7 Enrichment analysis of differential metabolic characteristics in kidney Yang deficiency type OP

**Figure 6:**
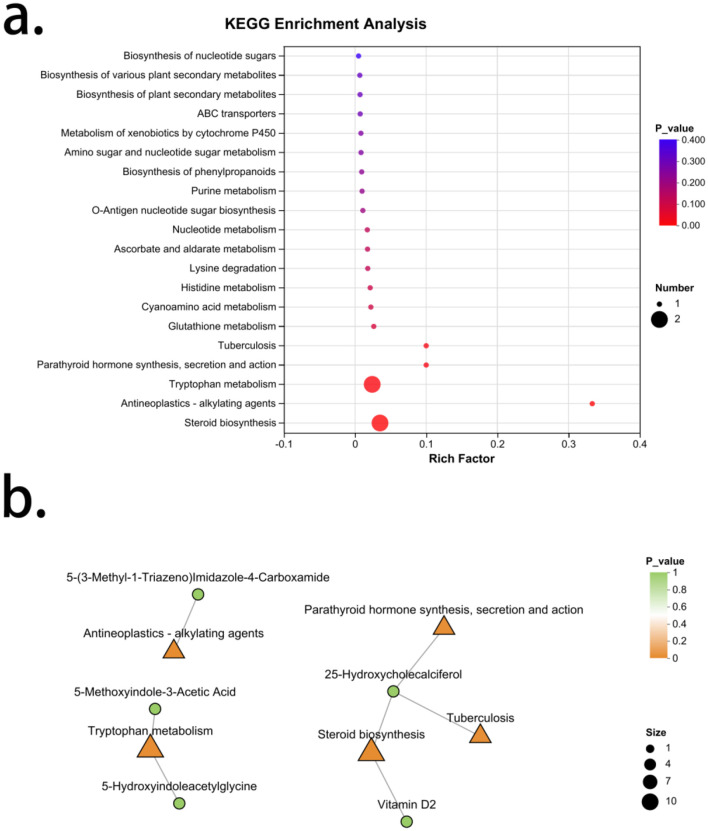
Enrichment analysis of characteristic metabolites in kidney Yang deficiency type OP. **(a)** KEGG enrichment analysis. **(b)** Gut microbiota-metabolite network.

A total of 20 signaling pathways were enriched from the differential metabolic characteristic set of kidney Yang deficiency type OP, with five pathways showing significance. Arranged in order of increasing *P*-values, these are: Steroid biosynthesis, Antineoplastics-alkylating agents, Tryptophan metabolism, Parathyroid hormone synthesis, secretion, and action and Tuberculosis (Figure 6a). The metabolites associated with steroid biosynthesis are Vitamin D2 and 25-Hydroxycholecalciferol, both of which are significantly reduced in kidney Yang deficiency type OP, indicating a decreased capacity for active vitamin D synthesis in this condition. The metabolite associated with Antineoplastics-alkylating agents is 5-(3-Methyl-1-Triazeno)Imidazole-4-Carboxamide, which is elevated in kidney Yang deficiency type OP. The compounds related to Tryptophan metabolism include 5-Methoxyindole-3-Acetic Acid and 5-Hydroxyindoleacetylglycine, both of which are significantly elevated in kidney Yang deficiency type OP. Additionally, the metabolites associated with Tuberculosis and the Parathyroid hormone synthesis, secretion, and action are both 25-Hydroxycholecalciferol (Figure 6b).

### 3.8 Enrichment analysis of differential metabolic characteristics in spleen-kidney Yang deficiency type OP

**Figure 7:**
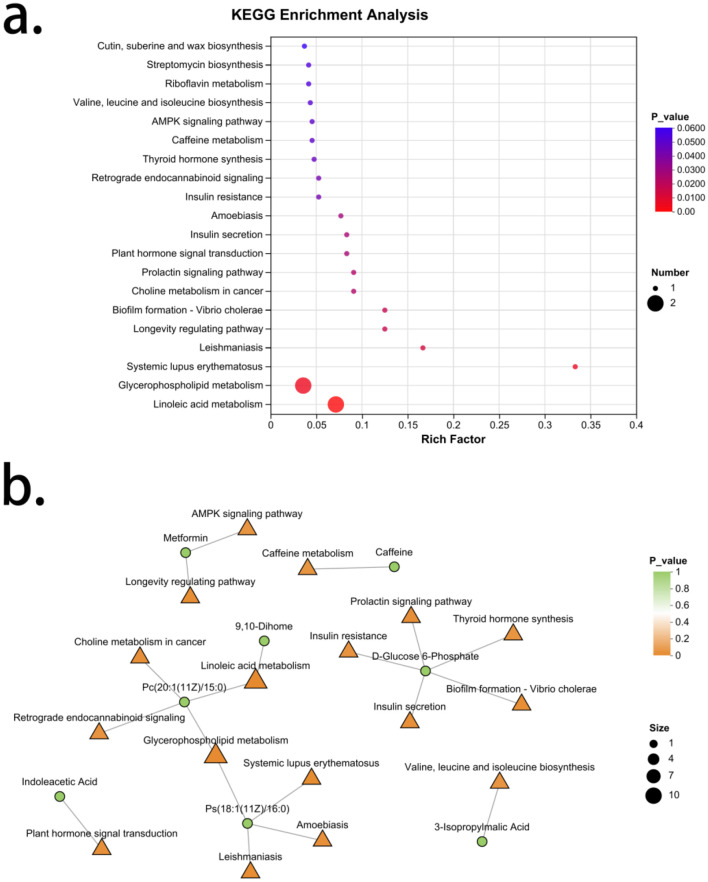
Enrichment analysis of characteristic metabolites in spleen-kidney Yang deficiency type OP. **(a)** KEGG enrichment analysis. **(b)** Gut microbiota-metabolite network.

An enrichment analysis of the differential metabolic characteristic set for spleen-kidney Yang deficiency type OP revealed a total of 44 signaling pathways, with 17 pathways showing significance. Arranged in order of increasing *P*-values, these are: Linoleic acid metabolism, Glycerophospholipid metabolism, Systemic lupus erythematosus, Leishmaniasis, Longevity regulation pathway, Biofilm formation—Vibrio cholerae, Choline metabolism in cancer, Prolactin signaling pathway, Plant hormone signal transduction, Insulin secretion, Amoebiasis, Retrograde endocannabinoid signaling, Insulin resistance, Thyroid hormone synthesis, Caffeine metabolism, AMPK signaling pathway, and Valine, leucine and isoleucine biosynthesis. The metabolites associated with Linoleic acid metabolism are Pc [20:1 (11Z)/15:0] and 9,10-Dihome, both of which are upregulated in spleen-kidney Yang deficiency type OP. The signaling pathway related to Pc [20:1 (11Z)/15:0] also includes Retrograde endocannabinoid signaling. The metabolites associated with Glycerophospholipid metabolism are Pc [20:1 (11Z)/15:0] and Ps [18:1 (11Z)/16:0], both of which are upregulated in spleen-kidney Yang deficiency type OP. The pathways related to Ps [18:1 (11Z)/16:0] also include Systemic lupus erythematosus, Leishmaniasis, and Amoebiasis. The metabolite associated with the Longevity regulation pathway is metformin, which is downregulated in spleen-kidney Yang deficiency type OP. The signaling pathway related to metformin also includes the AMPK signaling pathway. The metabolite associated with biofilm formation—Vibrio cholerae is D-Glucose 6-Phosphate, which is downregulated in spleen-kidney Yang deficiency type OP. Pathways related to D-Glucose 6-Phosphate also include choline metabolism in Choline metabolism in cancer signaling pathway, insulin secretion, insulin resistance, and thyroid hormone synthesis. The metabolite associated with plant hormone signal transduction is Indoleacetic Acid, which is upregulated in spleen-kidney Yang deficiency type OP. The metabolite related to caffeine metabolism is caffeine, which is downregulated in spleen-kidney Yang deficiency type OP. The metabolite associated with Valine, leucine and isoleucine biosynthesis is 3-Isopropylmalic Acid, which is also downregulated in spleen-kidney Yang deficiency type OP (Figure 7).

### 3.9 Analysis of the association between gut microbiota and metabolites

**Figure 8:**
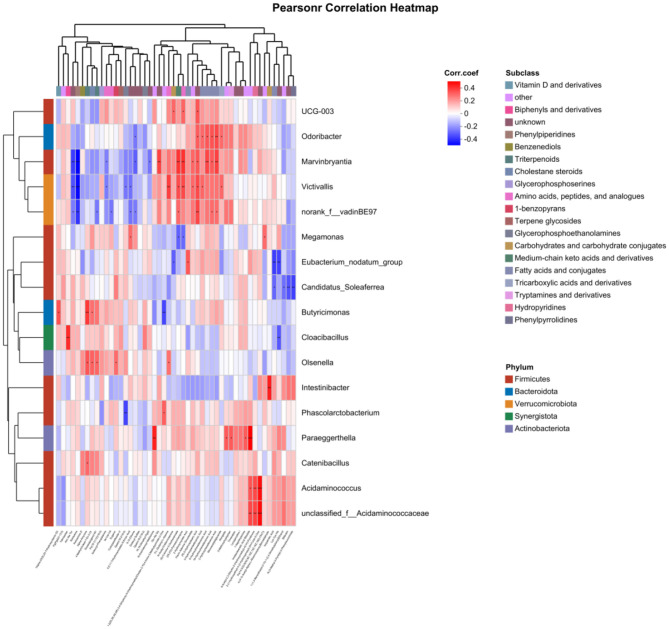
Correlation analysis between gut microbiota and metabolites.

A Pearson correlation analysis was conducted on the differential microbial taxa and differential metabolic characteristic sets for kidney Yang deficiency type and spleen-kidney Yang deficiency type OP. The results showed that the characteristic microbiota of kidney Yang deficiency type OP, specifically the genus Intestinibacter, was positively correlated with 4-(4′-O-Acetyl-Alpha-L-Rhamnosyloxy)Benzaldehyde (*r* = 0.41, *P* < 0.01). Meanwhile, the genus Phascolarctobacterium was negatively correlated with Gpetn (18:2/16:0; *r* = −0.37, *P* < 0.01) and positively correlated with 1- [(2S,3R,4S,5R)-3,4-Dihydroxy-5-(Hydroxymethyl)Oxolan-2-Yl]-4-Imino-3-Methylpyrimidin-2-One (*r* = 0.29, *P* < 0.05). For spleen-kidney Yang deficiency type OP, the characteristic microbiota genus Olsenella was positively correlated with Alpha-Amyrone (*r* = 0.31, *P* < 0.05), 4-Methylcholest-7-En-3-Ol (*r* = 0.29, *P* < 0.05), Soyasapogenol M1 (*r* = 0.28, *P* < 0.05), Eugenin (*r* = 0.27, *P* < 0.05), and 5-L-Glutamyl-L-Alanine (*r* = 0.26, *P* < 0.05; Figure 8).

## 4 Discussion

In the diagnosis and treatment of osteoporosis (OP) within traditional Chinese medicine (TCM), syndrome differentiation is emphasized, with spleen deficiency and kidney deficiency being central to therapeutic strategies. A growing body of evidence suggests that the material basis of “spleen–kidney” regulation is closely associated with the gut microbiota (Li et al., 2025; Wang et al., 2023; Ma et al., 2021). The gut microbiota, a complex ecosystem residing in the gastrointestinal tract, has its composition and diversity shaped by genetic factors and modulated by environmental influences. Its core functions include the regulation of host absorption, metabolism, and immune responses (Lyu et al., 2023), and it plays a pivotal role in the “spleen–muscle–bone” axis. Spleen deficiency can lead to reduced microbial diversity, decreased abundance of beneficial bacteria, and overgrowth of opportunistic pathogens. These alterations modulate muscle, bone, and adipose metabolism through multiple pathways, including effects on protein synthesis and metabolism, mitochondrial function, chronic inflammation, immune regulation, synthesis of short-chain fatty acids (SCFAs) and bile acids, production of harmful metabolites, and exosome-mediated intercellular communication (Geng et al., 2025). Although numerous studies have investigated microbial changes in OP, most indicate no significant alterations in overall microbial abundance or diversity (Huang et al., 2022). Our findings based on α- and β-diversity analyses are consistent with these reports. At the phylum level, *Firmicutes* and *Bacteroidota* predominated, and the F/B ratio—though associated with diet, energy metabolism, and dysbiosis—did not differ significantly between groups. Notably, the microbiota dysbiosis index (MDI), which reflects the degree of ecological imbalance, was significantly elevated in OP patients and most severe in those with spleen-kidney Yang deficiency, indicating profound microbial disruption in this subtype.

At the genus level, we identified 14 differentially abundant taxa and characterized signature genera in kidney Yang deficiency type OP and spleen-kidney Yang deficiency type OP. In kidney Yang deficiency type OP, *Intestinibacter* and *Phascolarctobacterium* were significantly enriched and negatively correlated with bone mineral density (BMD) across multiple sites. *Intestinibacter* has been proposed as an opportunistic pathogen closely linked to OP pathogenesis (Akinsuyi and Roesch, 2023). In contrast, *Phascolarctobacterium*, generally regarded as a beneficial SCFA producer, has also been reported to be enriched in OP (Ling et al., 2021), and its exact role in bone metabolism warrants further investigation. In spleen-kidney Yang deficiency type OP, *Olsenella* was significantly enriched and negatively correlated with appendicular skeletal muscle index (ASMI). *Olsenella* is a key genus associated with fat deposition (Zhang et al., 2024a) and obesity (Wei et al., 2024). Consistent with this, 33% of spleen-kidney Yang deficiency type OP patients had high body fat—significantly higher than the kidney Yang deficiency type OP group (13%)—helping explain the higher susceptibility to sarcopenia in spleen-kidney Yang deficiency type OP. MNA plays a key mediating role between *Olsenella* and spleen–kidney Yang deficiency, further demonstrating the adverse effects of *Olsenella* on nutrient absorption and metabolism in individuals with spleen–kidney Yang deficiency type OP. Furthermore, genera such as *Megamonas, Butyricimonas*, and *Victivallis*, which are associated with fatty acid metabolism and typically abundant in healthy populations, were reduced in OP and positively correlated with BMD or ASMI. These taxa facilitate carbohydrate metabolism to produce SCFAs, thereby promoting energy harvest and inhibiting bone resorption (Huang et al., 2022; Qin et al., 2021), underscoring the critical role of gut microbiota in OP pathogenesis.

Microbiota-derived metabolites significantly influence OP progression. Bile acids, key regulators of lipid metabolism, are closely linked to OP. Primary bile acids are converted by gut microbiota into secondary bile acids (e.g., 3-oxo-LCA and isoalloLCA), which affect the Th17/Treg balance by modulating reactive oxygen species (ROS) and inflammatory signaling, thereby regulating bone resorption (Qiao et al., 2022; Wen et al., 2020). Secondary bile acids can also act as vitamin D receptor ligands, regulating the metabolism of 1,25-dihydroxyvitamin D3 and playing a key role in skeletal homeostasis (Bouillon et al., 2014; Chaudhari et al., 2021). vitamin D2 and 25-hydroxycholecalciferol were significantly reduced in kidney Yang deficiency type OP, indicating impaired activation of vitamin D and compromised calcium absorption and utilization (Zhang et al., 2023). The downregulation of D-glucose 6-phosphate in spleen-kidney Yang deficiency type OP and its close association with insulin resistance and insulin secretion pathways suggest a significant coupling disorder of glucose and lipid metabolism in patients with spleen-kidney Yang deficiency. Notably, the significant upregulation of phosphatidylcholine derivative Pc [20:1 (11Z)/15:0] and phosphatidylserine Ps [18:1 (11Z)/16:0] indicates marked alterations in membrane lipid structure and abnormal lipid-mediated signaling under spleen-kidney Yang deficiency. Furthermore, the elevated levels of the polyunsaturated fatty acid metabolite 9,10-Dihome suggest that inflammatory lipid mediators may contribute to the progression of osteoporosis, aligning with the TCM theory of “phlegm-turbidity obstructing the interior” leading to bone wilting (osteoporosis). It is evident that osteoporosis with spleen-kidney Yang deficiency is closely associated with disordered lipid metabolism.

Other metabolites such as indole derivatives including serotonin (5-HT) inhibit bone formation through the gut–bone axis (Yan et al., 2018), whereas polyamines such as spermine and spermidine improve bone metabolism by enhancing bone strength (Feng et al., 2019; Yoshimoto et al., 2021). 5-Methoxyindole-3-acetic acid and 5-hydroxyindoleacetylglycine were upregulated in kidney Yang deficiency type OP, and indoleacetic acid was elevated in spleen-kidney Yang deficiency type OP—these may influence muscle and bone metabolism via tryptophan and serotonin pathways. Phytoestrogens including soybean isoflavones and their metabolite equol inhibit osteoclast activity by upregulating the osteoprotegerin (OPG)/RANKL ratio (Ni et al., 2023). Dietary polyphenols such as anthocyanins are metabolized by gut microbiota into bioactive compounds that suppress oxidative stress, reduce bone resorption, and promote bone formation (Wan et al., 2021).

The gut microbiota and these metabolites coregulate the dynamic balance of bone metabolism through a complex interaction network. *Olsenella* was positively correlated with several metabolites—α-amyrone, 4-methylcholest-7-en-3-ol, soyasapogenol M1, eugenin, and 5-L-glutamyl-L-alanine—all of which are triterpenes, sterols, or phenolic compounds with anti-inflammatory and antioxidant properties. However, the negative correlation between *Olsenella* and ASMI complicates its functional interpretation: it may either contribute to muscle loss in spleen-kidney Yang deficiency type OP or arise as a negative feedback response to sarcopenia. Given its role in promoting adiposity, *Olsenella* is likely a driver of muscle reduction and increased fat deposition, while the increase in anti-inflammatory and antioxidant metabolites may represent a compensatory mechanism.

In summary, this study clarifies the characteristics of gut microbiota and metabolites in different TCM subtypes of OP and explores their interrelationships. Both kidney Yang deficiency type and spleen-kidney Yang deficiency type OP exhibit gut microbiota dysbiosis, with the latter being more severe. The core signature taxa in kidney Yang deficiency type OP may be *Intestinibacter* and *Phascolarctobacterium*, whereas *Olsenella* may characterize spleen-kidney Yang deficiency type OP. More importantly, microbial metabolism in kidney Yang deficiency type OP is associated with vitamin D metabolism, while spleen-kidney Yang deficiency type OP is closely linked to disrupted lipid metabolism. These findings suggest that distinct TCM syndromes may correspond to unique microbial and metabolic pathophysiological pathways, providing novel insights into the modern chemical and biological basis of TCM syndrome differentiation.

## Data Availability

The data presented in this study are deposited in the NCBI BioProject repository (https://www.ncbi.nlm.nih.gov/bioproject), accession number PRJNA1338060.
