# Supplementary material for: Characterization of the integrated gut microbiota and metabolite profiles in osteoporosis patients with different traditional Chinese medicine syndromes
Source: Front Microbiol. 2025 Oct 22;16:1663716. doi: 10.3389/fmicb.2025.1663716 (PMC12587299; doi:10.3389/fmicb.2025.1663716)
Supplement: Supplementary file 2 [file Supplementary_file_2.docx]

**Supplementary Methods and Materials**

**1. DNA extraction and PCR amplification**

Total microbial genomic DNA was extracted from human fecal samples using the FastPure Stool DNA Isolation Kit (MJYH, shanghai, China) according to manufacturer’s instructions. The quality and concentration of DNA were determined by 1.0% agarose gel electrophoresis and a NanoDrop® ND-2000 spectrophotometer (Thermo Scientific Inc., USA) and kept at -80 ℃ prior to further use. The hypervariable region V3-V4 of the bacterial 16S rRNA gene were amplified with primer pairs 338F (5'-ACTCCTACGGGAGGCAGCAG-3') and 806R(5'-GGACTACHVGGGTWTCTAAT-3') by an T100 Thermal Cycler (BIO-RAD, USA). The PCR reaction mixture including 4 μL 5 × Fast Pfu buffer, 2 μL 2.5 mM dNTPs, 0.8 μL each primer (5 μM), 0.4 μL Fast Pfu polymerase, 10 ng of template DNA, and ddH2O to a final volume of 20 µL. PCR amplification cycling conditions were as follows: initial denaturation at 95 ℃ for 3 min, followed by 27 cycles of denaturing at 95 ℃ for 30 s, annealing at 55 ℃ for 30 s and extension at 72 ℃for 45 s, and single extension at 72 ℃ for 10 min, and end at 4 ℃. All samples were amplified in triplicate. The PCR product was extracted from 2% agarose gel and purified. Then quantified using Synergy HTX (Biotek, USA).

**2. Illumina sequencing**

Purified amplicons were pooled in equimolar amounts and paired-end sequenced on an Illumina NextSeq 2000 PE300 platform (Illumina, San Diego, USA) according to the standard protocols by Majorbio Bio-Pharm Technology Co. Ltd. (Shanghai, China).

**3. Data processing**

Raw FASTQ files were de-multiplexed using an in-house perl script, and then quality-filtered by fastp version 0.19.6 and merged by FLASH version 1.2.11 with the following criteria: (i) the PE300 reads were truncated at any site receiving an average quality score of <20 over a 50 bp sliding window, and the truncated reads shorter than 50 bp were discarded, reads containing ambiguous characters were also discarded; (ii) only overlapping sequences longer than 10 bp were assembled according to their overlapped sequence. The maximum mismatch ratio of overlap region is 0.2. Reads that could not be assembled were discarded; (iii) Samples were distinguished according to the barcode and primers, and the sequence direction was adjusted, exact barcode matching, 2 nucleotide mismatch in primer matching. Then the optimized sequences were clustered into operational taxonomic units (OTUs) using Usearch 11 with 97% sequence similarity level. The most abundant sequence for each OTU was selected as a representative sequence. ,To minimize the effects of sequencing depth on alpha and beta diversity measure, the number of 16S rRNA gene sequences from each sample were rarefied to 37553, which still yielded an average Good’s coverage of 99.09%, respectively.

The taxonomy of each OTU representative sequence was analyzed by RDP Classifier version 2.13 against the 16S rRNA gene database (eg. Silva v138) using confidence threshold of 0.7.

**4. Gut Microbiota Data Analysis**

Bioinformatic analysis of the gut microbiota was carried out using the Majorbio Cloud platform (https://cloud.majorbio.com). Based on the OTUs information, rarefaction curves and alpha diversity indices including observed OTUs, Chao1 richness, Shannon index and Good’s coverage were calculated with Mothur v1.30.2. The similarity among the microbial communities in different samples was determined by principal coordinate analysis (PCoA) based on Bray-curtis dissimilarity using Vegan v2.4.3 package. The PERMANOVA test was used to assess the percentage of variation explained by the treatment along with its statistical significance using Vegan v2.4.3 package. The linear discriminant analysis (LDA) effect size (LEfSe) (http://huttenhower.sph.harvard.edu/LEfSe) was performed to identify the significantly abundant taxa (phylum to genera) of bacteria among the different groups (LDA score > 2, P < 0.05).

**5. Metabolite Extraction**

100 mg solid sample was added to a 2 mL centrifuge tube and a 6 mm diameter grinding bead was added. 800 μL of extraction solution (methanol: water = 4:1 (v:v) containing four internal standards (0.02 mg/mL L-2-chlorophenylalanine, etc.) were used for metabolite extraction. Samples were ground by the Wonbio-96c (Shanghai wanbo biotechnology co., LTD) frozen tissue grinder for 6 min (-10°C, 50 Hz), followed by low-temperature ultrasonic extraction for 30 min (5°C, 40 kHz). The samples were left at-20°C for 30 min, centrifuged for 15 min (4°C, 13000 g), and the supernatant was transferred to the injection vial for LC-MS/MS analysis.

**6. Quality control sample**

As a part of the system conditioning and quality control process, a pooled quality control sample (QC) was prepared by mixing equal volumes of all samples. The QC samples were disposed and tested in the same manner as the analytic samples. It helped to represent the whole sample set, which would be injected at regular intervals (every 5-15 samples) in order to monitor the stability of the analysis.

**7. UHPLC-MS/MS analysis**

The LC-MS/MS analysis of sample was conducted on a UHPLC-Q Exactive system equipped with an ACQUITY HSS T3 column (100 mm × 2.1 mm i.d., 1.8 μm; Waters, USA) at Majorbio Bio-Pharm Technology Co. Ltd. (Shanghai, China). The mobile phases consisted of 0.1% formic acid in water:acetonitrile (2:98, v/v) (solvent A) and 0.1% formic acid in acetonitrile (solvent B). The flow rate was 0.40 mL/min and the column temperature was 40℃. The injection volume was 5 μL.

MS conditions: The UHPLC system was coupled to a UHPLC-Q Exactive system Mass Spectrometer equipped with an electrospray ionization (ESI) source operating in positive mode and negative mode. The optimal conditions were set as followed: source temperature at 400℃ ; sheath gas flow rate at 40 arb; Aux gas flow rate at 10 arb; ion-spray voltage floating (ISVF) at-2800V in negative The detection was carried out over a mass range of 70-1050 m/z.

**8. Data preprocessing and annotation**

The pretreatment of LC/MS raw data was performed by Progenesis QI (Waters Corporation，

Milford, USA) software, and a three-dimensional data matrix in CSV format was exported. The information in this three-dimensional matrix included: sample information, metabolite name and mass spectral response intensity. Internal standard peaks, as well as any known false positive peaks (including noise, column bleed, and derivatized reagent peaks), were removed from the data matrix, deredundant and peak pooled. At the same time, the metabolites were identified by searching database, and the main databases were the HMDB (http://www.hmdb.ca/), Metlin ( https://metlin.scripps.edu/) and the self-compiled Majorbio Database (MJDB) of Majorbio Biotechnology Co., Ltd. (Shanghai, China) The data matrix obtained by searching database was uploaded to the Majorbio cloud platform (https://cloud.majorbio.com) for data analysis. Firstly, the data matrix was pre-processed, as follows: At least 80% of the metabolic features detected in any set of samples were retained. After filtering, the minimum value in the data matrix was selected to fill the missing value and each metabolic signature was normalized to the sum. To reduce the errors caused by sample preparation and instrument instability, the response intensities of the sample mass spectrometry peaks were normalized using the sum normalization method, to obtain the normalized data matrix. Meanwhile, the variables of QC samples with relative standard deviation (RSD) > 30% were excluded and log10 logarithmicized, to obtain the final data matrix for subsequent analysis. Perform variance analysis on the matrix file after data preprocessing.

The R package “ropls”(Version 1.6.2) was used to perform principal component analysis (PCA) and orthogonal least partial squares discriminant analysis (OPLS-DA), and 7-cycle interactive validation evaluating the stability of the model. The metabolites with VIP>1, p<0.05 were determined as significantly different metabolites based on the Variable importance in the projeciton (VIP) obtained by the OPLS-DAmodel and the p-value generated by student’s t test. Differential metabolites among two groups were mapped into their biochemical pathways through metabolic enrichment and pathway analysis based on KEGG database (http://www.genome.jp/kegg/). These metabolites could be classified according to the pathways they involved or the functions they performed. Enrichment analysis was used to analyze a group of metabolites in a function node whether appears or not. The principle was that the annotation analysis of a single metabolite develops into an annotation analysis of a group of metabolites. Python packages “scipy.stats” (https://docs.scipy.org/doc/scipy/ ) was used to perform enrichment analysis to obtain the most relevant biological pathways for experimental treatments.
